# Utility of MR lymphangiography in chylous ascites: A report of two cases

**DOI:** 10.4102/sajr.v27i1.2703

**Published:** 2023-08-31

**Authors:** Pratibha Bhatia, Poonam Sherwani, Intezar Ahmed, Udit Chauhan, Sudhir Saxena

**Affiliations:** 1 Department of Radiodiagnosis and Imaging, All India Institute of Medical Science, Rishikesh, India; 2 Department of Pediatric Surgery, All India Institute of Medical Science, Rishikesh, India

**Keywords:** MR lymphangiography, central conducting lymphatics, chylous ascites, nodal lymphangiography, cisterna chyli

## Abstract

Dynamic contrast-enhanced (DCE) lymphangiography is a novel imaging technique with a potential role in suspected cases of lymphatic leaks. A 15-year-old male with a post operative chylous leak and an 8-year-old male who developed chylous ascites secondary to disseminated tuberculosis are presented. Both children underwent MR lymphangiography.

**Contribution:**

The role of DCE-MR lymphangiography in cases of chylous ascites to help guide appropriate management.

## Introduction

Imaging of the lymphatic system in children may be indicated in cases of suspected lymphatic disorders or lymphatic leaks. However, given the complex network of lymphatic channels and their small size, developments in imaging of the lymphatic system have trailed behind.

Conventional fluoroscopic intranodal or pedal lymphangiography using ethiodol-based contrast agents was performed earlier for the diagnosis of lymphatic disorders, but places the risk of ionising radiation exposure in the paediatric population. Dynamic contrast-enhanced (DCE) MR lymphangiography following the injection of gadolinium-based agents into groin nodes is a novel technique that allows visualisation of the central conducting lymphatics (CCLs), including the retroperitoneal lymphatics, which is imperative in cases of chylous ascites or chylothorax.

This report describes two cases of chylous ascites where dynamic MR lymphangiography was performed at our centre after cannulation of bilateral groin nodes.

## Case report

### Case 1

A 15-year-old male developed abdominal pain and distension following blunt trauma to the abdomen, which was insidious in onset and gradually progressive. A week following the trauma, the patient presented to a nearby local hospital and ultrasound abdomen revealed gross free fluid in the peritoneal cavity and a pancreatic pseudocyst. The patient underwent exploratory laparotomy and pelvic drain insertion. Upon resuming an oral diet, there was a chylous output of approximately 500 mL – 600 mL per day from the drain and the patient was referred to our institute. Initially, endoscopic ultrasound was performed with a pancreatic cystogastrostomy and metallic stent placement. Oral intake was ceased and total parenteral nutrition and Octreotide infusion was commenced; however, the drain output persisted.

**FIGURE 1:**
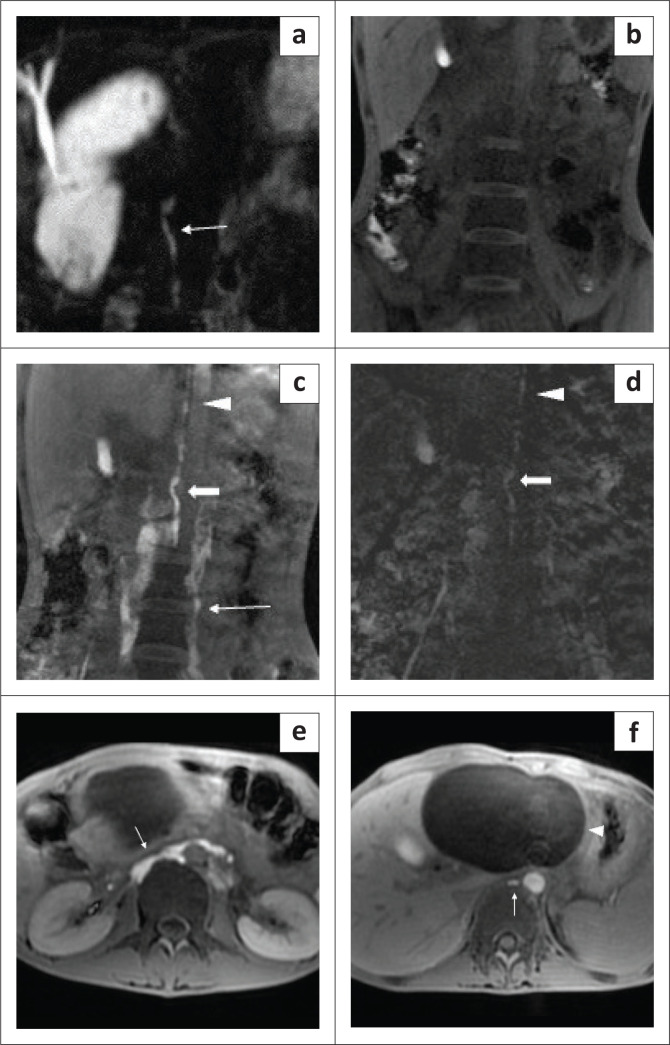
MR lymphangiography in a 15-year-old child with postoperative chylous ascites. The central conducting lymphatics were interpreted as normal in this child. (a) Coronal heavily T2-weighted MRI images of the abdomen with thin Maximum Intensity Projection (MIP) reconstruction depicted a normal cisterna chyli (white arrow). (b) Coronal pre-contrast T1-weighted image of abdomen and pelvis and (c) coronal T1-weighted image of abdomen and pelvis at 7 min after nodal contrast injection demonstrated normal opacification of the cisterna chyli (bold arrow) with faint opacification of the thoracic duct (arrow head). Contrast was still visible in the lumbar lymphatics (arrow). No intraperitoneal leak of contrast was seen. (d) The central conducting lymphatics were highlighted in the subtracted image depicting the cisterna chyli (bold arrow) and thoracic duct (arrow head). (e) Axial T1-weighted image 5 min after intranodal contrast administration showed confluence of the bilateral lumbar lymphatics to form the cisterna chyli (arrow) at the level of renal hilum. (f) Axial T1-weighted image of the upper abdomen 9 min after intranodal contrast administration depicted a normal thoracic duct (arrow). A pancreatic pseudocyst (arrow head) was seen anterior to it.

A DCE-MR lymphangiogram study was performed for the patient after intranodal gadolinium administration. A normal calibre and course of cisterna chyli was seen on coronal heavily T2-weighted images (Figure 1a). Post nodal contrast scans revealed the right and left lumbar lymphatic channels, joining to form the cisterna chyli (Figure 1e), followed by opacification of the thoracic duct on subsequent images (Figure 1c, d and f). No major duct injury was seen. There was no contrast extravasation in the peritoneal cavity.

**FIGURE 2:**
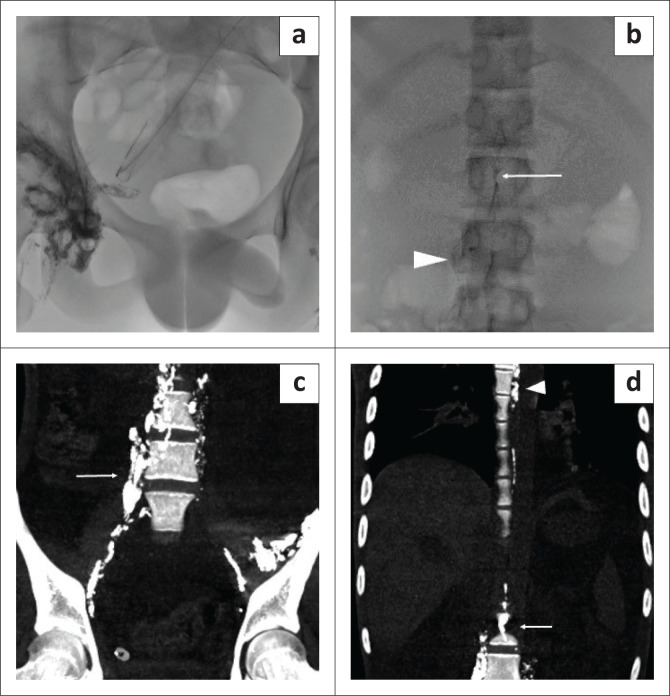
Fluoroscopic nodal lymphangiography was performed in the same child for diagnostic and therapeutic purposes. (a) Fluoroscopic images showed contrast injected in bilateral groin nodes. (b) Fluoroscopic image 30 min after intranodal lipiodol injection depicted a normal cisterna chyli (arrow) and lumbar lymphatics (arrowhead). No intraperitoneal contrast leak was seen. Coronal reformatted CT images post fluoroscopic lymphangiography with thin MIP reconstruction of the abdomen and pelvis (c) and chest (d) demonstrated normal opacification of the lumbar lymphatics (arrow in c) and normal opacification of the cisterna chyli (arrow in d) and thoracic duct (arrowhead in d).

Nodal fluoroscopic lymphangiography was also performed for the patient a few days after the MRI study. Following the administration of lipiodol through the inguinal nodes, multiple spot images were obtained of the pelvic and retroperitoneal lymphatics (Figure 2a and b). No free spill was seen in the abdominal cavity. Post-procedural CT also revealed normal anatomy of the centrally conducting lymphatic channels without contrast extravasation (Figure 2c and d).

The likely site for the lymphatic leak was attributed to the distal intestinal lymphatic channels without any injury to the central conducting lymphatics. The patient proceeded to surgery and underwent lipiodol injection into the mesenteric lymph nodes. Following surgery, the drain output gradually reduced, and the patient showed clinical improvement.

### Case 2

**FIGURE 3:**
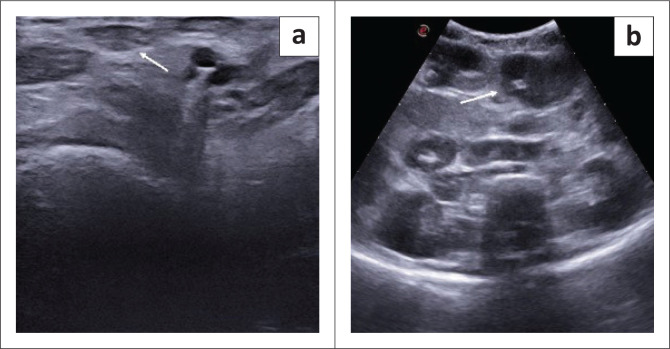
Ultrasound of the groin (a) and abdomen (b) in an 8-year-old male with disseminated tuberculosis showed (a) enlarged inguinal nodes (arrow) and (b) multiple enlarged abdominal lymph nodes (arrow).

An 8-year-old male presented with complaints of high-grade fever, weight loss and abdominal distension for 1–2 months. Ultrasonography revealed hepatomegaly with multiple hypoechoic liver nodules, enlarged necrotic lymph nodes (Figure 3a and b) and gross ascites. A clinical diagnosis of disseminated tuberculosis was suggested. Biopsy from the mesenteric lymph nodes confirmed tuberculous lymphadenitis. Ascitic fluid analysis revealed elevated triglyceride content suggestive of chylous ascites.

**FIGURE 4:**
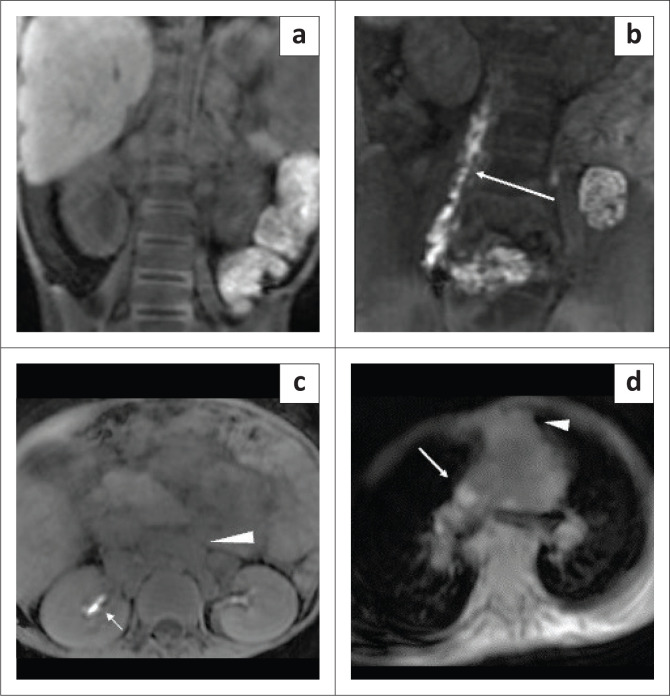
Dynamic contrast enhanced MR lymphangiography was performed in the same child for chylous ascites. (a) Coronal pre-contrast T1-weighted image of the abdomen and pelvis and (b) coronal T1-weighted image of the abdomen and pelvis at 10 min after nodal contrast injection showed dilated lumbar lymphatics (arrow) and non-opacification of the central conducting lymphatics. (c) Axial T1-weighted image of the abdomen 30 min after intranodal contrast administration showed non-opacification of the central conducting lymphatics. Contrast opacification was noted in the renal pelvis. Enlarged abdominal lymph nodes could also be seen (arrow head). (d) Axial T1-weighted image of the chest 40 min after intranodal contrast administration showed non-opacification of the thoracic duct in the mediastinum but contrast opacification was visible in the superior vena cava (arrow). Enlarged lymph nodes were seen in the anterior mediastinum (arrow head).

**FIGURE 5:**
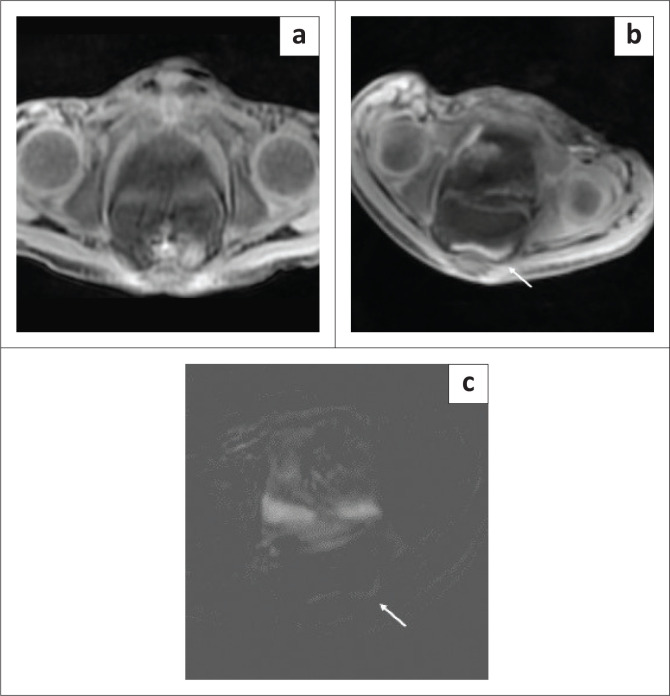
Case 2 (a) axial pre-contrast T1-weighted image of the pelvis and (b) axial T1-weighted image of the pelvis 12 min after intranodal contrast administration depicted leakage of contrast in the pelvic free fluid (arrow). (c) The leak was highlighted in the subtracted image (arrow).

Dynamic contrast enhanced MR lymphangiography was performed after ultrasound guided cannulation of the inguinal lymph nodes. Dilute gadolinium-based contrast was injected on the MRI table. The scan revealed dilated bilateral lumbar lymphatics that were opacified within a few minutes of contrast injection with non-opacification of the cisterna chyli (Figure 4b). Contrast accumulation was seen in the pelvic free fluid on subsequent images (Figure 5). The CLLs remained unopacified on delayed phase imaging due to compression by enlarged retroperitoneal lymph nodes (Figure 4c and d).

## Discussion

Chylous ascites is the accumulation of milky triglyceride-rich fluid in the peritoneal cavity. It occurs because of disruption or obstruction of the lymphatic ducts leading to extravasation of lymphatic fluid.^1^ In adults, cirrhosis and malignancy are the most common causes, while in childhood, trauma and iatrogenic injury are more often the underlying aetiologies. Among infective causes, tuberculosis is an important aetiological factor.^1^

In the presented scenarios, the first child presented with milky drain output after resuming feeds post-surgery likely due to intra-operative injury to the lymphatic channels. The second was a case of disseminated tuberculosis with chylous ascites, likely because of obstruction of lymphatic flow secondary to enlarged lymph nodes causing compression and increased back pressure with resultant lymphatic fluid extravasation. It is important to delineate the site of disruption or obstruction and alert the surgeon or radiologist to guide management. It is also imperative to discern whether or not the central conducting lymphatics are involved as they are less likely to respond to conservative management.^2,3,4^

Imaging of the lymphatic system has been difficult due to the complex nature and small size of the lymphatics.^5^ Earlier, fluoroscopic lymphangiography techniques using ethiodol-based contrast agents were used for both diagnostic and therapeutic purposes. However, the relationship of the lymphatic duct with adjacent structures cannot be delineated, thus limiting the role of fluoroscopic-guided lymphangiographic techniques to therapeutic interventions. More recently, the use of DCE-MR lymphangiography with gadolinium-based contrast agents helps overcome the limitations of fluoroscopic techniques.

Pedal lymphangiography techniques involve the injection of contrast into the interdigital space in the foot and the absorption of contrast from the interstitium into the lymphatic channels.^5,6,7^ Intranodal lymphangiographic techniques utilise injection of contrast into the groin lymph nodes.^8,9^ Peripheral or pedal MR lymphangiography is an older and simpler technique, but visualisation of the retroperitoneal and central conducting lymphatics is difficult when using these techniques.^5^

In both presented cases, intranodal DCE-MR lymphangiography was performed. The procedure involved initial routine MRI abdomen and pelvis sequences using a 3 tesla MRI scanner along with heavily T2-weighted images for delineation of the cisterna chyli and thoracic duct. Following this, the patient was shifted outside the MRI unit for nodal cannulation under ultrasound guidance. Using an aseptic technique, 1% lidocaine was injected intradermally at the area of interest, following which, bilateral superficial inguinal lymph nodes were cannulated using a 22-gauge needle followed by saline injection. The lymph nodes became more prominent after saline injection and after ensuring adequate placement, the needle was connected to 10-cm long tubing attached at its end to a three-way stop cock. A syringe containing a gadolinium and saline solution ratio of 1:1 was connected to the stop cock. The gadolinium dosage was 0.1 mmol/kg. The patient then returned to the MRI table and a mask T1-weighted image of the chest and abdomen was obtained prior to the injection of gadolinium via the stop cock. Multiple axial and coronal post-contrast T1-weighted (VIBE) images of the abdomen followed by the chest were obtained at every 1-min interval for about 30 minutes, and subtracted images were generated to enhance the sensitivity for detecting intraperitoneal contrast leakage.

Variable findings can be seen on MR lymphangiography depending on the aetiology of chylous ascites and the level of occlusion or leak, which may involve the intestinal lymphatics, cisterna chyli or lower thoracic duct. Extravasation of contrast into the peritoneal cavity may also be seen.^5^ Dynamic contrast-enhanced MR lymphangiography is more sensitive than radiographic lymphangiography in establishing the site of the leak because of the lower viscosity of gadolinium-based contrast agents compared to ethiodised oil. However, in more than half of the cases, the leak site may not be established. This is because the cisterna chyli receives supply from the intestinal, hepatic and bilateral lumbar lymphatics, and it is the lumbar lymphatics that receive direct drainage from the inguinal nodes while the intestinal lymphatics are off track and distant from the site of injection.^2,5^ However, establishing the integrity of the central conducting lymphatics is crucial for management decisions.

In the first case, there was no evidence of injury to the cisterna chyli or thoracic duct on MR lymphangiography. As a result, surgery was performed and embolising agents were injected into the mesenteric nodes to target the distal intestinal lymphatics. In the second case there was non-opacification of the CCLs cranial to the renal hilum due to compression by enlarged abdominal lymph nodes. The patient was commenced on anti-tuberculous therapy with close monitoring and there was a gradual reduction in chylous output.

## Conclusion

MR lymphangiography is a novel technique with a promising role in the management of patients with chylous ascites and chylous effusions. However, it is underutilised despite no extra cost or equipment requirement other than basic MRI and ultrasound facilities. More research is warranted with this imaging technique to consolidate its role in the management of lymphatic disorders, especially in the paediatric population.
